# Supplementary material for: Causal Relationship between Aging and Anorexia Nervosa: A White-Matter-Microstructure-Mediated Mendelian Randomization Analysis
Source: Biomedicines. 2024 Aug 16;12(8):1874. doi: 10.3390/biomedicines12081874 (PMC11351342; doi:10.3390/biomedicines12081874)
Supplement: Supplementary file 1 [file biomedicines-12-01874-s001.zip › Supplementary Figure/Figure S4/Singe SNP plot of MR analysis for TL to ubm-b-1668.pdf]

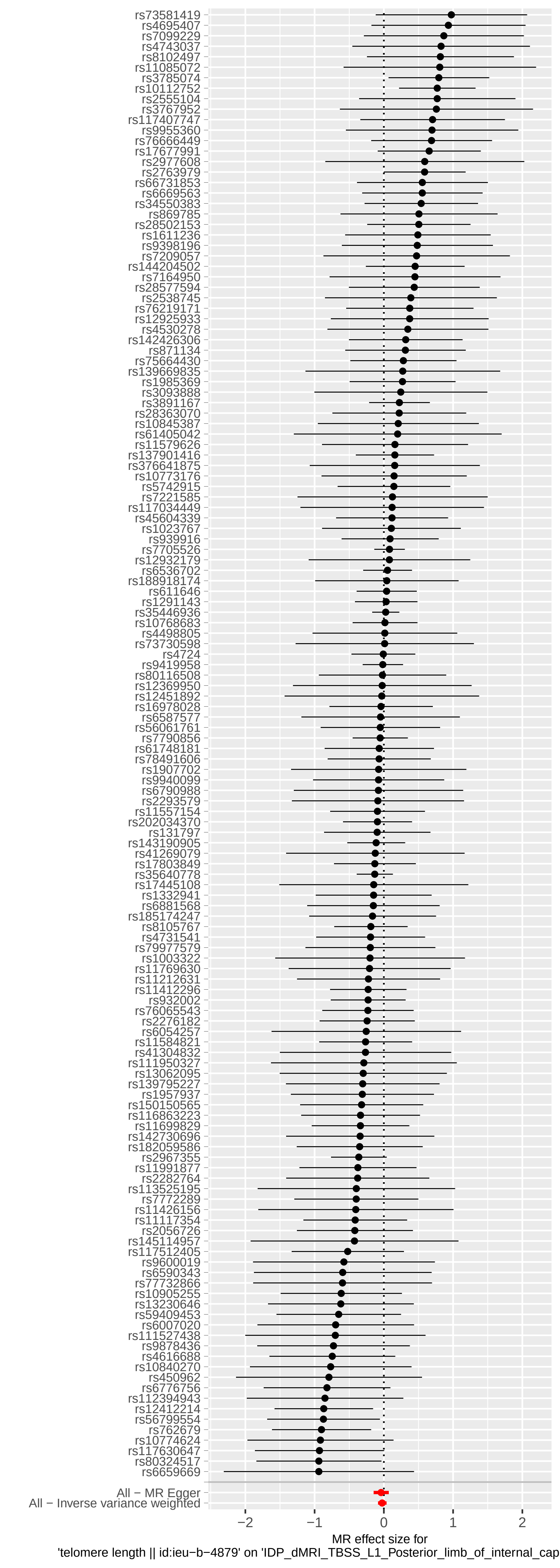
rs73581419
rs4695407
rs7099229
rs4743037
rs8102497
rs11085072
rs3785074
rs10112752
rs2555104
rs3767952
rs117407747
rs9955360
rs76666449
rs17677991
rs2977608
rs2763979
rs66731853
rs6669563
rs34550383
rs869785
rs28502153
rs1611236
rs9398196
rs7209057
rs144204502
rs7164950
rs28577594
rs2538745
rs76219171
rs12925933
rs4530278
rs142426306
rs871134
rs75664430
rs139669835
rs1985369
rs3093888
rs3891167
rs28363070
rs10845387
rs61405042
rs11579626
rs137901416
rs376641875
rs10773176
rs5742915
rs7221585
rs117034449
rs45604339
rs1023767
rs939916
rs7705526
rs12932179
rs6536702
rs188918174
rs611646
rs1291143
rs35446936
rs10768683
rs4498805
rs73730598
rs4724
rs9419958
rs80116508
rs12369950
rs12451892
rs16978028
rs6587577
rs56061761
rs7790856
rs61748181
rs78491606
rs1907702
rs9940099
rs6790988
rs2293579
rs11557154
rs202034370
rs131797
rs143190905
rs41269079
rs17803849
rs35640778
rs17445108
rs1332941
rs6881568
rs185174247
rs8105767
rs4731541
rs79977579
rs1003322
rs11769630
rs11212631
rs11412296
rs932002
rs76065543
rs2276182
rs6054257
rs11584821
rs41304832
rs111950327
rs13062095
rs139795227
rs1957937
rs150150565
rs116863223
rs11699829
rs142730696
rs182059586
rs2967355
rs11991877
rs2282764
rs113525195
rs7772289
rs11426156
rs11117354
rs2056726
rs145114957
rs117512405
rs9600019
rs6590343
rs77732866
rs10905255
rs13230646
rs59409453
rs6007020
rs111527438
rs9878436
rs4616688
rs10840270
rs450962
rs6776756
rs112394943
rs12412214
rs56799554
rs762679
rs10774624
rs117630647
rs80324517
rs6659669
All − MR Egger
All − Inverse variance weighted
−2
−1
0
1
2
MR effect size for
'telomere length || id:ieu−b−4879' on 'IDP_dMRI_TBSS_L1_Posterior_limb_of_internal_cap
